# Psychological well-being in elderly adults with extraordinary episodic memory

**DOI:** 10.1371/journal.pone.0186413

**Published:** 2017-10-23

**Authors:** Amanda Cook Maher, Stephanie Kielb, Emmaleigh Loyer, Maureen Connelley, Alfred Rademaker, M.-Marsel Mesulam, Sandra Weintraub, Dan McAdams, Regina Logan, Emily Rogalski

**Affiliations:** 1 Cognitive Neurology & Alzheimer’s Disease Center, Northwestern University Feinberg School of Medicine, Chicago, Illinois, United States of America; 2 Department of Psychiatry & Behavioral Sciences, Northwestern University Feinberg School of Medicine, Chicago, Illinois, United States of America; 3 Department of Preventive Medicine, Northwestern University Feinberg School of Medicine, Chicago, Illinois, United States of America; 4 Department of Neurology, Northwestern University Feinberg School of Medicine, Chicago, Illinois, United States of America; 5 Foley Center for Lives, Northwestern University, Evanston, Illinois, United States of America; University of Akron, UNITED STATES

## Abstract

**Objectives:**

The Northwestern University SuperAging Program studies a rare cohort of individuals over age 80 with episodic memory ability at least as good as middle-age adults to determine what factors contribute to their elite memory performance. As psychological well-being is positively correlated with cognitive performance in older adults, the present study examined whether aspects of psychological well-being distinguish cognitive SuperAgers from their cognitively average-for-age, same-age peers.

**Method:**

Thirty-one SuperAgers and 19 cognitively average-for-age peers completed the Ryff 42-item Psychological Well-Being questionnaire, comprised of 6 subscales: Autonomy, Positive Relations with Others, Environmental Mastery, Personal Growth, Purpose in Life, and Self-Acceptance.

**Results:**

The groups did not differ on demographic factors, including estimated premorbid intelligence. Consistent with inclusion criteria, SuperAgers had better episodic memory scores. Compared to cognitively average-for-age peers, SuperAgers endorsed greater levels of Positive Relations with Others. The groups did not differ on other PWB-42 subscales.

**Discussion:**

While SuperAgers and their cognitively average-for-age peers reported similarly high levels of psychological well-being across multiple dimensions, SuperAgers endorsed greater levels of positive social relationships. This psychological feature could conceivably have a biological relationship to the greater thickness of the anterior cingulate gyrus and higher density of von Economo neurons previously reported in SuperAgers.

## Introduction

Age-related cognitive decline is widely considered to be a “normal” consequence of aging. However, evidence from the Northwestern University SuperAging Program suggests that such decline is not inevitable [1]. SuperAgers are adults over age 80 whose performance on tests of episodic memory, the type of memory that shows decline with aging and dramatic decline in Alzheimer’s dementia, is at least as good as individuals 20 to 30 years their junior. The SuperAging Program is dedicated to identifying factors that contribute to their elite memory performance.

Initial results suggest that SuperAgers represent a rare cognitive phenotype with unique physiological brain features. SuperAgers are selected for having above-average-for-age episodic memory at the time of study entry and most maintain this high level of performance over at least 18-months, suggesting resistance to age-related memory decline [2]. On structural magnetic resonance imaging (MRI) scans at the time of study entry, SuperAgers had a significantly thicker brain cortex than their cognitively average, same-age peers and did not show significant cortical thinning compared to cognitively average middle-age adults [3]. SuperAgers also had a significantly larger left anterior cingulate than both the cognitively average-for-age elderly and middle-age groups [3]. This cortical preservation is unusual as age-related cortical atrophy is well documented beginning in early adulthood [4, 5]. Further, a recent longitudinal neuroimaging study in the SuperAging Program found that SuperAgers demonstrate significantly less whole-brain cortical atrophy over 18-months compared to their cognitively average, same-age peers [6]. While the functional significance of the difference in cortical thickness and difference in atrophy rates is currently unclear, these neuroimaging findings contribute to the hypothesis that SuperAgers may possess unique brain physiology.

This idea is furthered by post-mortem histopathologic analyses of the anterior cingulate, which demonstrated a lower frequency of Alzheimer disease pathology and a higher density of von Economo neurons (VENs) in SuperAgers compared to their cognitively average same-age peers [7]. VENs are a type of neuron that have been identified in humans and other phylogenetically advanced species, are primarily found in the anterior cingulate and frontoinsular cortices, and are hypothesized to play a role in complex social cognition, intuitive assessment of complex, often uncertain, situations, and facilitation of fast communication with other brain regions [8, 9]. Taken together, these findings suggest that SuperAgers have unique biological characteristics that separate them from their cognitively average-for-age peers, which may contribute to their resistance to age-related involutional changes in memory [1, 7]. The present study extends these initial findings by characterizing psychological well-being in SuperAgers.

Psychological well-being is a positive psychological construct related to subjective views of one’s self and life. Higher levels of psychological well-being have been positively correlated with cognitive performance in older adults. For example, Llewellyn and colleagues (2008) demonstrated that among individuals over age 50, those who scored in the highest quintile on the CASP-19 psychological well-being scale scored higher on neuropsychological tests than those who scored in the lowest quintile [10]. Previous research has also shown positive correlations between episodic memory performance and environmental mastery [11], purpose in life [12, 13], and positive relations with others as characterized by social support and number of social contacts [14, 15] in older adults. In addition, greater baseline levels of purpose in life [12] and increased social engagement later in life [16, 17] have both been associated with reduced risk of developing Alzheimer’s dementia, a neurodegenerative disease characterized by primary impairment in episodic memory. Because SuperAgers are selected for having superior episodic memory for their age, it is possible that they also express greater environmental mastery, purpose in life, and positive relations with others compared to their cognitively average-for-age same-age peers. The present study therefore examined whether aspects of psychological well-being distinguish cognitively above-average SuperAgers from their cognitively average-for-age peers.

## Methods

### Participants

Data were prospectively collected from participants enrolled in the Northwestern University SuperAging Program, an ongoing, longitudinal investigation of aging in community-dwelling adults over age 80 who have been described previously [1]. Briefly, SuperAgers are carefully differentiated from cognitively average-for-age, same-age elderly adults based on neuropsychological test performance as shown in **Table 1**. Inclusion criteria for SuperAgers is as follows: 1) performance at or above normative values for *average 50 to 65-year-olds* (equivalent to the Superior range for their own age) on delayed recall of the Rey Auditory Verbal Learning Test (RAVLT), a 15-word list-learning episodic memory test [18]; 2) performance in at least the average-for-age normative range on non-memory tests including the 30-item Boston Naming Test [19], Trail Making Test Part-B [20], and Category Fluency Test [21]. In contrast, inclusion criteria for Cognitively Average Elderly adults is as follows: 1) performance within the average-for-age normative range on episodic memory (i.e. the RAVLT); 2) performance within the average-for-age normative range on all non-memory tests listed above.

**Table 1:** Inclusion criteria for SuperAgers and cognitively average elderly adults.

	SuperAger	Cognitively Average Elderly Adult
**Age**	≥80 years	≥80 years
**Episodic Memory Performance**	Average for 50–65 year old	Average for ≥80 year old
**Non-memory Cognitive Performance**	At least average for ≥80 year old	Average for ≥80 year old

All participants were required to have preserved activities of daily living and lack clinical evidence of significant neurological or psychiatric illness. In the present study, participants were required to maintain their cognitive status (SuperAger or cognitively average elderly) from enrollment to the time questionnaires were completed to minimize the unintended inclusion of individuals with emerging Alzheimer’s dementia and maintain the integrity of our elite SuperAger sample.

The Northwestern University Institutional Review Board approved study procedures. All participants provided written informed consent.

### Questionnaire

The Ryff 42-item Psychological Well Being questionnaire (PWB-42; [22, 23] was used to assess psychological well-being. The PWB-42 is a self-report measure that includes six domains of well-being: Environmental Mastery, Personal Growth, Positive Relations with Others, Self-Acceptance, Autonomy, and Purpose in Life, (described in **Table 2)**. This six-factor model has support for use in healthy elderly adults [22, 24]. Participants rate their agreement with 42 statements on a 6-point Likert scale ranging from “1 –strongly disagree” to “6 –strongly agree.” Scores in each domain range from 7 and 42 where higher scores indicate greater levels of well-being. One-half of items are negatively worded and reverse scored.

**Table 2:** Ryff & Keyes psychological well-being 42-item questionnaire subscale descriptions[22, 23]. Examples of positively worded and negatively worded items are provided.

PWB-42 Subscales	Definition & Sample Item
Autonomy	Independence, self-determination; ability to resist social pressures. *“I have confidence in my opinions*, *even if they are contrary to the general consensus*.*” “It is difficult for me to voice my own opinions on controversial matters*.*”*
Positive Relations with Others	Satisfying, warm, trusting, high-quality relationships with others. *“I know that I can trust my friends and they know they can trust me*.*” “I often feel lonely because I have few close friends with whom to share my concerns*.*”*
Environmental Mastery	Mastery and competence in managing one’s life and environment. *“I am quite good at managing the many responsibilities of my daily life*.*” “I often feel overwhelmed by my responsibilities*.*”*
Personal Growth	Feeling of continued development; being open to new experiences. *“It is important to have new experiences that challenge how you think about yourself and the world*.*” “There is a truth in the saying that you can’t teach an old dog new tricks*.*”*
Purpose in Life	Belief that one’s life is meaningful; aims and objectives for living. *“I have a sense of direction and purpose in life*.*” “My daily activities often seem trivial and unimportant to me*.*”*
Self-Acceptance	Positive attitude towards, and acceptance of, one’s self and past. *“When I look at the story of my life*, *I am pleased with how things have turned out*.*” “My attitude about myself is probably not as positive as most people feel about themselves*.*”*

### Statistical analysis

Because the subscale scores were not normally distributed in SuperAgers, Wilcoxon rank sum tests and Fisher’s exact tests were used to examine between-group differences in demographic variables, neuropsychological data, and PWB-42 subscale scores, as appropriate. Wilcoxon rank sum test was also used to examine potential gender differences on the PWB-42 subscales. Spearman correlations were used to examine the association between episodic memory performance and psychological well-being. All tests were two-tailed.

For each set of six rank sum and correlation tests for PWB-42 subscales, a Bonferroni criterion of p<0.0083 was used for statistical significance. With the given sample sizes, there was 80% power for the rank sum test to detect a mean difference of 4.3 in the PWB subscales between groups at the adjusted p<0.0083.

## Results

### Demographic & neuropsychological characterization

Thirty-one SuperAgers (ages 80 to 96, 23 female) and 19 Cognitively Average Elderly adults (ages 80 to 102, 12 female) were actively enrolled in the SuperAging Program, met inclusion criteria, and completed the PWB-42 questionnaire. All are included in this analysis.

There were no significant group differences in age, gender, race, or education (*p*’s>0.05; **Table 3**). Consistent with inclusion criteria, SuperAgers had significantly better RAVLT performance compared to their cognitively average peers (median delay raw score (interquartile range (IQR)) SuperAgers: 11 (10–13); Cognitively Average Elderly: 7 (6–7)); *p*<0.001). The groups did not differ on other neuropsychological measure, including estimates of premorbid intellectual ability (*p*’s>*0*.*05*).

**Table 3:** Demographic & neuropsychological characteristics of the sample. There were no significant between-group differences in age, education, gender, or race. SuperAgers outperformed their cognitively average-for-age peers on measures of episodic memory and category fluency. There were no other significant between-group differences on neuropsychological measures, including estimated premorbid intelligence.

	SuperAgers *n = 31*	Cognitively Average Elderly *n = 19*	p-value
**Age (years)** *Median (IQR)*	83.4 (81.7–85.4)	84.4 (81.7–86.3)	0.43
**Education** (**years)** *Median (IQR)*	16 (14–18)	18 (16–18)	0.18
**Gender** *(Percentage Female)*	74.2%	63.2%	0.53
**Race Ratio** *(Percentage White)*	96.8%	89.5%	0.55
**Estimated Premorbid I.Q.** *WTAR Median (IQR)*	118 .0(114–122)	117.5 (109–121)	0.63
Episodic Memory* *RAVLT Delay Raw Score Median (IQR) (Maximum Possible Score is 15)*	11 (10–13)	7 (6–7)	<0.0001
**Object Naming** *BNT-30 Raw Score Median (IQR) (Maximum Possible Score is 30)*	29 (27–29)	28 (26–29)	0.14
**Category Fluency** *‘Animal’ Raw Score Median (IQR)*	22 (18–26)	20 (15–23)	0.11
**Executive Attention** *TMT-B Total Time Median (IQR)*	78 (63–102)	96.5 (73–117)	0.14

IQR: Interquartile range, WTAR: Wechsler Test of Adult Reading Estimated Full-Scale Intelligence Quotient, RAVLT: Rey Auditory Verbal Learning Test, BNT-30: 30-item Boston Naming Test, TMT-B: Trail Making Test Part-B.

* Indicates a significant between-group difference

### Psychological well-being

**Fig 1:**
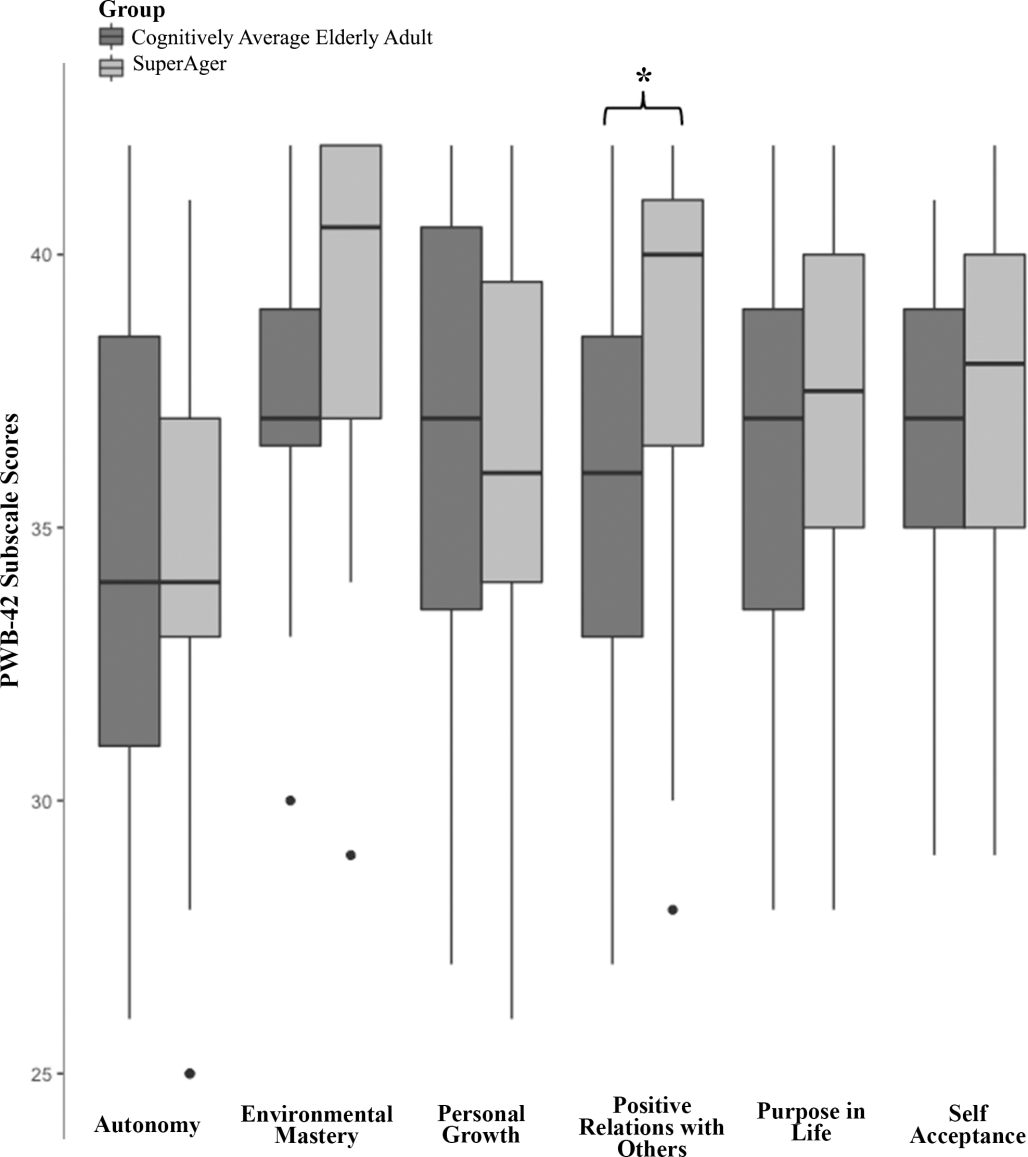
Psychological well-being in SuperAgers and cognitively average-for-age elderly adults. Boxplots for Psychological Well-Being 42-Item (PWB-42) subscale scores are shown (medians, interquartile range, and upper/lower quartile ranges). SuperAgers endorsed significantly greater levels of positive relations with others compared to their cognitively average peers (Median SuperAger: 40, Cognitively Average Elderly: 36; *p* = 0.005). The groups did not differ on other subscales of the PWB-42 (*p’s*>0.0083) including Autonomy (Median SuperAger: 34, Cognitively Average Elderly: 34), Environmental Mastery (Median SuperAger: 40.5, Cognitively Average Elderly: 37), Personal Growth (Median SuperAger: 36, Cognitively Average Elderly: 37), Purpose in Life (Median SuperAger: 37.5, Cognitively Average Elderly: 37), or Self-Acceptance (Median SuperAger: 38, Cognitively Average Elderly: 37).

Scores from the PWB-42 are shown in **Fig 1**. SuperAgers endorsed a significantly greater level of Positive Relations with Others (median overall score (IQR): 40 (36–41); mean item score: 5.5) compared to Cognitively Average Elderly adults (median overall score (IQR): 36 (33–39); mean item score: 5.1; *p* = 0.005). There were no other significant between-group differences on PWB-42 subscales (*p*’s>*0*.*0083*). There were no significant gender differences within either group on the PWB-42 subscales (*p*-values ranged from 0.14 to 0.94).

Spearman correlation showed low positive associations between RAVLT performance and Environmental Mastery (Spearman r = 0.36, *p* = 0.013) and between RAVLT performance and Positive Relations with Others (Spearman r = 0.33, *p* = 0.018), but were not significant with Bonferroni correction.

## Discussion

While both SuperAgers and Cognitively Average Elderly adults endorsed high levels of psychological well-being, SuperAgers endorsed greater levels of positive social relationships than their cognitively average-for-age peers suggesting that perceived high-quality social relationships may be an important factor in cognitive SuperAging. This result is particularly intriguing given the previously reported higher density of von Economo neurons found in the anterior cingulate cortex of SuperAgers [1, 7] and the hypothesized role of these neurons in higher order social cognition and behaviors, such as social bonding, social intuition, and emotional regulation [8, 9, 25]. It is possible that the greater density of these neurons in SuperAgers underlies increased positive social interactions and behaviors, which in turn contribute to greater endorsement of Positive Relations with Others on the PWB-42. As von Economo neurons can only be quantified via histopathological analysis post-mortem, the relationship between von Economo neuronal density and PWB-42 endorsement remains speculative.

Our finding that SuperAgers endorse greater levels of positive social relationships than their cognitively average-for-age peers is consistent with previous research demonstrating that social integration, engagement with family, and emotional support from a social network are positively correlated with cognitive function in older adults [15, 26, 27], predict reduced rates of cognitive decline [14, 28], and are associated with reduced risk of Mild Cognitive Impairment and dementia [29, 30]. Importantly, it appears that social relationships in and of themselves are important to the maintenance of cognition as opposed to other factors that may enhance or inhibit participation in social relationships, such as sex, depressive symptoms, baseline cognitive ability, physical functioning, or social network size [16]. In addition, the relationship between positive social relationships and cognition appears to be unidirectional in that positive social relationships influence subsequent cognitive performance while the reverse does not appear to be true [31]. Because SuperAgers represent a rare cognitive aging phenotype and sample sizes are therefore small compared to other psychology studies, current findings should be validated in larger, more demographically diverse samples.

SuperAgers and the cognitively average-for-age elderly adults in this study shared similar levels of Autonomy, Environmental Mastery, Personal Growth, Purpose in Life, and Self-Acceptance. This suggests that these aspects of psychological well-being may not differ among cognitively average to superior adults. On average, both our cognitively average adults and SuperAgers endorse slightly higher levels of well-being than the general elderly population studied in MIDUS (Midlife in the United States: a National Longitudinal Study of Health and Well-being); [32], suggesting both groups in the present study may be on the higher end of the psychological well-being spectrum. However, there is a lack of literature with published average values for adults over age 80 on the PWB-42 limiting our ability to compare findings from the SuperAgers and cognitively average-for-age elderly with other populations. The correlation between episodic memory performance and Positive Relations with Others was not significant in our sample, suggesting there may not be a direct relationship among elderly individuals with average to superior memory.

The presence of gender differences in measures of psychological well-being are inconsistent across studies, with some studies showing that women tend to rate themselves higher on Positive Relations [33, 34] and others finding that men may rate themselves higher on Personal Growth [33] or Purpose in Life [12]. The present study did not find gender differences on any measure of psychological well-being, although the study was not specifically designed for such comparison.

In summary, SuperAgers endorse higher levels of Positive Relations with Others compared to their cognitively healthy but average-for-age same-age peers suggesting that this aspect of psychological well-being may be an important factor for exceptional cognitive aging. Investigation of the longitudinal effects of psychological well-being on subsequent cognitive performance and investigation of the conceivable relationship between psychological well-being and von Economo neurons in SuperAgers represent intriguing future directions.

## Supporting information

S1 File Supporting information. This file contains the raw data used for analyses.
(XLSX)
